# Assessing Language Lateralization through Gray Matter Volume: Implications for Preoperative Planning in Brain Tumor Surgery

**DOI:** 10.3390/brainsci14100954

**Published:** 2024-09-24

**Authors:** Daniel Solomons, Maria Rodriguez-Fernandez, Francisco Mery-Muñoz, Leonardo Arraño-Carrasco, Francisco Sahli Costabal, Carolina Mendez-Orellana

**Affiliations:** 1 Institute for Biological and Medical Engineering, Schools of Engineering, Medicine and Biological Sciences, Pontificia Universidad Católica de Chile, Santiago 7820436, Chile; dsolomons@uc.cl (D.S.); marodriguezf@uc.cl (M.R.-F.); fsc@ing.puc.cl (F.S.C.); 2 Millennium Institute for Intelligent Healthcare Engineering—iHEALTH, Santiago 7820436, Chile; 3 Department of Neurosurgery, Faculty of Medicine, Pontificia Universidad Católica de Chile, Santiago 7820436, Chile; franciscomery@neurocirugiauc.cl; 4 Department of Radiology, Faculty of Medicine, Pontificia Universidad Católica de Chile, Santiago 7820436, Chile; larrano@gmail.com; 5 Department of Mechanical and Metallurgical Engineering, School of Engineering, Pontificia Universidad Católica de Chile, Santiago 7820436, Chile; 6 Speech and Language Pathology Department, Health Sciences School, Faculty of Medicine, Pontificia Universidad Católica de Chile, Santiago 7820436, Chile

**Keywords:** brain tumor, gray matter volume, language lateralization, regression analysis

## Abstract

Background/Objectives: Functional MRI (fMRI) is widely used to assess language lateralization, but its application in patients with brain tumors can be hindered by cognitive impairments, compensatory neuroplasticity, and artifacts due to patient movement or severe aphasia. Gray matter volume (GMV) analysis via voxel-based morphometry (VBM) in language-related brain regions may offer a stable complementary approach. This study investigates the relationship between GMV and fMRI-derived language lateralization in healthy individuals and patients with left-hemisphere brain tumors, aiming to enhance accuracy in complex cases. Methods: The MRI data from 22 healthy participants and 28 individuals with left-hemisphere brain tumors were analyzed. Structural T1-weighted and functional images were obtained during three language tasks. Language lateralization was assessed based on activation in predefined regions of interest (ROIs), categorized as typical (left) or atypical (right or bilateral). The GMV in these ROIs was measured using VBM. Linear regressions explored GMV-lateralization associations, and logistic regressions predicted the lateralization based on the GMV. Results: In the healthy participants, typical left-hemispheric language dominance correlated with higher GMV in the left pars opercularis of the inferior frontal gyrus. The brain tumor participants with atypical lateralization showed increased GMV in six right-hemisphere ROIs. The GMV in the language ROIs predicted the fMRI language lateralization, with AUCs from 80.1% to 94.2% in the healthy participants and 78.3% to 92.6% in the tumor patients. Conclusions: GMV analysis in language-related ROIs effectively complements fMRI for assessing language dominance, particularly when fMRI is challenging. It correlates with language lateralization in both healthy individuals and brain tumor patients, highlighting its potential in preoperative language mapping. Further research with larger samples is needed to refine its clinical utility.

## 1. Introduction

Functional MRI (fMRI) has become the preferred tool for mapping language regions and determining language lateralization [1]. Making this determination is specifically relevant in clinical contexts, where the treatment of conditions such as brain tumors, epilepsy, and vascular malformations relies on the accurate determination of the regions responsible for language processing [2].

Language lateralization is routinely measured through fMRI in patients with diverse neurological conditions [3,4,5], and it can be categorized as left, right, or bilateral. However, the use of fMRI in mapping language regions includes various practical limitations. Language activation depends on the task performance inside the scanner, which, regarding patients with cognitive or language impairments, may result in long or unsuccessful scanning sessions due to the tasks’ demanding nature. For example, brain tumor and stroke patients may present problems completing the scanning protocols [5]. Furthermore, language lateralization varies according to the linguistic domain and task modality, reflecting the intrinsic variability in brain function rather than being solely a result of the fMRI paradigm used [6,7,8]. This variability is an inherent characteristic of functional lateralization, influenced by factors such as the nature of the task and the subject’s cognitive state. Consequently, while fMRI provides valuable insights, there remains a need for alternative noninvasive methods that can reliably determine language lateralization in clinical populations, particularly those with severe cognitive and language impairments. This is especially important in low- and middle-income countries in which access to MRI scanning is limited [9], and patients’ cognitive performance deteriorates as they remain on waiting lists to access neuroimaging evaluation or surgical intervention.

Language lateralization is calculated through a lateralization index (LI) derived from fMRI data [7,8,10,11]. The accuracy of the results of an LI can be affected by the threshold selected in its calculation and, in the case of clinical groups, the fMRI artifacts introduced by the brain lesions [12,13,14]. Given these difficulties, visual inspection of activation patterns by a skilled neuroradiologist is preferred in clinical contexts [8,15,16], alongside verifying crossed cerebrocerebellar activation as a marker of lateralization [8,10].

An alternative neuroimaging modality to determine language lateralization is voxel-based morphometry (VBM), which can be used to assess the gray matter volume (GMV) across the brain [17,18,19,20]. Clinical studies have demonstrated associations between GMV and fMRI-based language activity in patients with epilepsy and brain tumors, as well as in healthy participants. However, these associations have primarily been observed with resting-state fMRI rather than task-based fMRI, and they are notably limited to regions such as the cerebellum [21,22]. In the presence of a brain tumor, plasticity effects are evident in language regions such as Broca’s and Wernicke’s areas, as detected by both functional measures (e.g., task-based fMRI) and structural measures (e.g., VBM) [23,24].

The approach of this study, which involves associating fMRI task-based language activity with GMV, is unproven in brain tumor patients in the language network regions, and it not only complements the conventional task-based fMRI method but also offers a new, comprehensive, and multimodal perspective of language lateralization, with a specific focus on clinically significant scenarios. The tasks undertaken by the participants and patients during fMRI scanning include verbal fluency (VG), phonological association (PA), and semantic association (SA) tasks, all of which have been established in clinical assessments of language lateralization [8,25,26]. In the VG task, the participants covertly think of a verb associated with a presented noun while their brain activity is recorded; however, this task’s covert nature means that performance cannot be directly observed or quantified, which can be challenging, especially for individuals with aphasia [8,25]. In contrast, the PA task requires the participants to press a button when two words rhyme, and the SA task involves pressing a button when two words share a meaning. These latter tasks are more straightforward and provide clearer measures of language processing through overt responses.

Our tasks have been clinically validated and were specifically designed to be suitable for this population [27]. Additionally, the tasks were designed to target the CCC (crossed cerebrocerebellar activation) and were later validated against VG [10]. These validations support the effectiveness and appropriateness of the tasks for assessing language lateralization in clinical settings.

The aim of this study is to establish a direct and validated connection between GMV and functional language lateralization using healthy and clinical data, as confirmed by assessments from neuroradiologists. An additional novelty of this study is the potential establishment of GMV as a predictive factor for functional lateralization, a dimension that was previously unexplored [21]. Overall, the current study has the potential to significantly advance our insights into language lateralization, offering instrumental contributions to the optimization of presurgical strategies and patient care.

## 2. Materials and Methods

### 2.1. Participants

We retrospectively collected MRI data between July 2016 and July 2018 on participants from outpatient clinics of the departments of neurosurgery at the Hospital Barros Luco Trudeau, Hospital Complejo Asistencial Dr. Sótero Del Rio, and Hospital Clínico Pontificia Universidad Católica de Chile. The cohort included 22 healthy participants (HPs) with a mean age 41.32 years, SD = 16.29, female = 12, male = 10, right-handed = 16, and left-handed = 6. Additionally, 28 participants with left-sided brain tumors (participant mean age 44.3 years, SD = 15.5, female = 17, male = 11, right-handed = 27, and left-handed = 1) were included, 13 of which were previously reported in previous research [8]. The handedness of participants was determined using the Edinburgh Handedness Inventory in Spanish, a validated measure of handedness [28]. Tumors were primary neoplasms classified by biopsy as glioblastoma, oligodendroglioma, astrocytoma, or tuberculoma. Tumor grade, location, and volume, as well as further demographic data of the brain tumor group, are available in Supplementary Table S1. Overall demographic information and statistical comparisons of the demographic data of the participant groups are available in Table 1.

Inclusion criteria were native Spanish speaker, monolingual, and aged 18 to 75. Exclusion criteria included history of language or speech disorders, severe hearing deficits, significant visual perceptual disorders, severe motor disabilities, recent psychiatric history, and contraindications for MRI. None were under observation for psychiatric disorders and were not taking psychopharmacological medication; however, records of long-term previous psychological disorders were not assessed. For brain tumor participants, additional criteria required a single left-hemisphere lesion, no prior tumor surgery, and a language deficit that did not preclude participation in language fMRI tasks. Although formal language tests were not administered, inclusion and exclusion criteria ensured that participants could engage in fMRI language tasks. Participants earmarked for surgery were not undergoing concurrent chemotherapy or radiotherapy. The study protocol was approved by the scientific medical ethical committee (Approval N° 15-302), Pontificia Universidad Católica de Chile and Servicio de Salud Metropolitano Sur, and written informed consent was obtained from all participants.

### 2.2. MRI Acquisition

Participants were scanned using a Philips Ingenia 3-T MR system. A volumetric T1-weighted structural image (echo time = 3023.2 ms; repetition time = 8.2 ms) with an effective slice thickness of 1.0 mm was acquired for anatomical registration and, in the case of those in the brain tumor participant group, to assess the localization and size of the lesion. Functional images were acquired using a gradient echo-planar imaging pulse-weighted T2* sequence with echo time = 30 ms as three different language tasks were completed (VG, PA, and SA). The repetition time for the VG task was 3000 ms; for the PA and SA tasks, it was 3500 ms.

### 2.3. Functional MRI Language Tasks and Language Lateralization Determination

**Figure 1:**
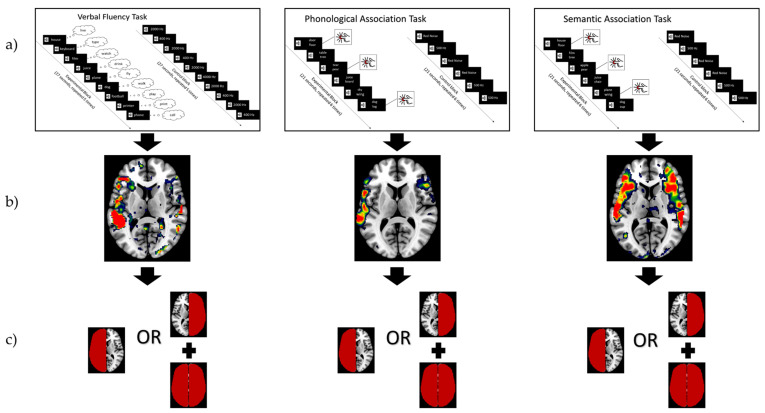
How task-based fMRI was used to create the language lateralization score, which was compared to GMV in statistical analyses. (**a**) Participants performed the verbal fluency (VG), phonological association (PA), and semantic association (SA) tasks inside the MRI scanner. (**b**) fMRI BOLD signal was recorded during task completion and was analyzed by neuroradiologists, with the color red displaying higher BOLD activity. (**c**) The neuroradiologist score was either typical (left-dominant fMRI BOLD activity, displayed in red) or atypical (right-dominant or bilateral fMRI BOLD activity, displayed in red).

Whilst in the MRI scanner, participants completed three language tasks, the verbal fluency (VG), semantic association (SA), and phonological association (PA) tasks (Supplementary Figure S1), following a previously conducted procedure [8]. Functional imaging data were pre-processed using SPM, version 12 all run within MATLAB version R2023a. Data from individual subjects were spatially realigned, co-registered to their respective high-resolution structural image, and smoothed. A general linear model was used to generate statistical activation maps to visualize activation related to task performance. Functional MRI activation, in the form of individual t-contrast images during each task, was examined by an expert neuroradiologist, blinded to the language task conducted and viewed at a threshold-independent level, in accordance with previous work [10]. To determine functional language lateralization, the activations were inspected at the following language regions of interest (ROIs): the inferior frontal gyrus: pars opercularis (IFG-op), pars triangularis (IFG-tri), and pars orbitalis (IFG-orb); and superior temporal gyrus (STG), middle temporal gyrus (MTG), supplementary motor area (SMA), angular gyrus (AG), and the supramarginal gyrus (SMG). Crossed cerebrocerebellar activation was additionally analyzed [8,10,29]. Activation was then categorized as either left-lateralized, right lateralized, bilateral, or no activation. Additionally, a second neuroradiologist verified the scores. Lastly, in accordance with the previous literature, language lateralization was categorized as “typical” when activation was in the left hemisphere, and bilateral and right-sided activation were categorized as “atypical” [30]. The typical/atypical split is based on population-based studies showing that 96% of right-handed and 70% of left-handed people are functionally left-lateralized during language function [31]. Figure 1 displays how participation in fMRI tasks (Figure 1a) led to recording of fMRI activity (Figure 1b), which was then assigned a binary lateralization score by a neuroradiologist (Figure 1c).

### 2.4. ROI-Based Morphometry Analysis

**Figure 2:**
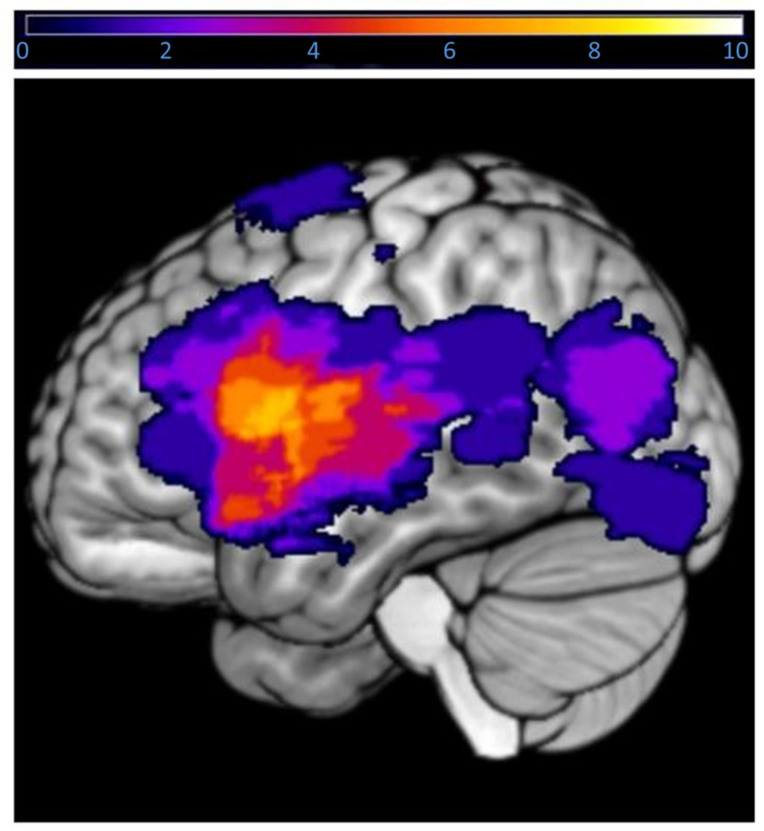
Tumor location and overlap of the 28 participants included in the tumor patient analysis. The color bar displays how many participants have a lesion in the area.

Structural T1 images were segmented into gray matter (GM), white matter (WM), and cerebrospinal fluid (CSF), with a stroke lesion correction using binary tumor masks to remove it during segmentation process using the CAT12 toolbox in SPM12. By including these masks in the segmentation, created by two expert neuroradiologists in MRIcroN (version 1.0.20190902) using the T1 image in the native space of each individual participant [32], the tumor is not included during the classification of brain tissue. Figure 2 displays the locations of the tumors. The segmented images were normalized to the MNI space; however, GMV values of an individual participant’s original T1 scan, known as their “native space”, were extracted by warping the normalized T1 image back into the participant’s native space, together with a personalized atlas parcellating the brain into different ROIs [33]. In this case, the “Neuromorphometrics” atlas was used, and GMV was extracted from 8 ROIs. As the GMV within the ROI was in a native, non-normalized space that is variable depending on participants’ head size, all statistical analyses in this study used total intercranial volume (TIV) as a control variable [34]. GMV values were extracted from the same language ROIs inspected by the neuroradiologist for scoring functional language lateralization [8,10,28]. See Supplementary Figure S2 for a visualization of the ROIs selected over the MNI152 T1 MRI template. The ROI masks were created with the WFU Pickatals aal brain atlas on SPM12 and visualized with MRIcroGL software, version 1.2.20210317 [35,36]. In brain tumor participants, only the right hemisphere was analyzed due to the disruption in T1 signal caused by the presence of the left-hemisphere tumor in the surrounding areas. The unit for the measurement of tumor and TIV was set to milliliters (mL).

### 2.5. Statistical Analyses

Linear regressions were conducted comparing functional language lateralization during each task (VG, PA, and SA) and GMV values from our selected language ROIs. These analyses were performed separately in the healthy participant group and left-hemisphere brain tumor group. The covariates were TIV, age, and tumor volume in the latter group to ensure that GMV differences caused by size, age, and the size of the tumor were accounted for. Due to the high volume of regressions being conducted, a false discovery rate (FDR) correction, the Benjamini and Hochberg method, was applied to correct for multiple comparisons [37]. When significant association after FDR correction was found between GMV (calculated in our language ROI) and functional language lateralization during the linear regression, a binomial logistic regression was conducted to test whether the GMV could predict functional language lateralization. Statistical analyses were run in MATLAB, and visualizations of the logistic regressions were created using Jamovi^®^ software, version 2.2.5 [38].

## 3. Results

### 3.1. Demographic Comparisons

Table 1, which includes *t*-tests comparing various demographic factors between the functionally typical and atypical groups, shows how handedness was not statistically related to whether a participant was typically or atypically lateralized. This trend was also observed for sex, age, and tumor volume in the tumor patient group. Total intercranial volume (TIV) was significantly higher in the typical group during the PA task; however, it was included as a covariate in all the statistical analyses along with age, and tumor volume when applicable, when analyzing the relationship between fMRI lateralization and GMV. Overall, the analysis suggests that sex, age, and handedness did not influence the functional lateralization of the participants during this analysis.

### 3.2. Linear Regression

#### 3.2.1. Healthy Participants

**Figure 3:**
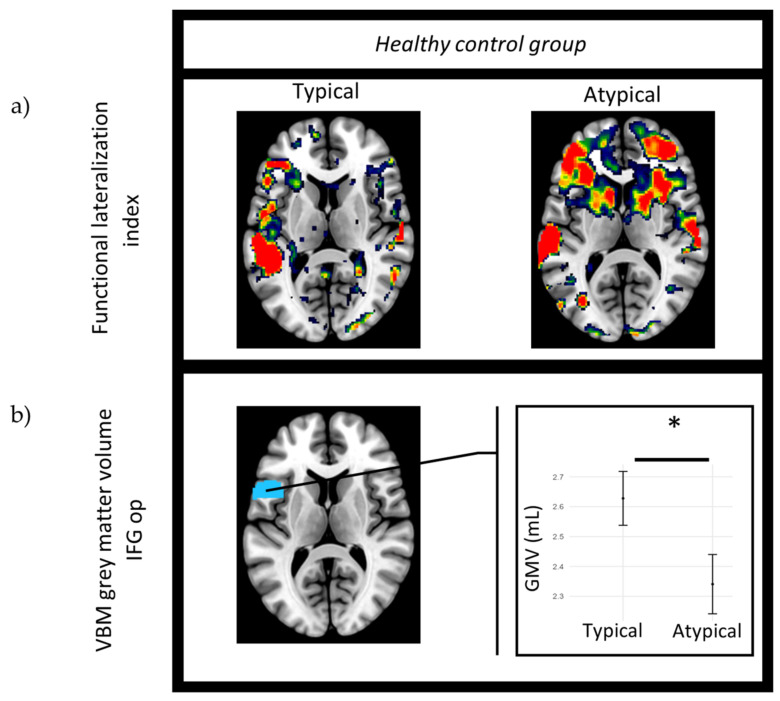
Healthy control group: (**a**) functional language lateralization during the semantic association (SA) task for typical participant (subject 12) and atypical participant (subject 6). Higher activity (red) is observed in the left hemisphere of the typical participant, with high activity seen in both hemispheres in the atypical participant. (**b**) Statistical GMV differences between functionally typical and atypical healthy participants during the SA task in the left opercular inferior frontal gyrus (IFG op). * = *p* ≤ 0.05 after FDR correction.

During the SA task, in the typically lateralized participants, the left hemisphere of the IFG op displayed a higher volume, shown in Table 2. Figure 3a,b show how more functional activity in the left hemisphere of healthy patients overlapped with higher GMV in the left IFG op. Out of the three language tasks, the SA task was the only one that separated the typical and atypical healthy participants following the FDR correction, showing a higher left IFG op in the typically lateralized participants. No regions showed significantly higher GMV in the atypical group compared to the typical group.

#### 3.2.2. Participants with Brain Tumor in the Left Hemisphere

**Figure 4:**
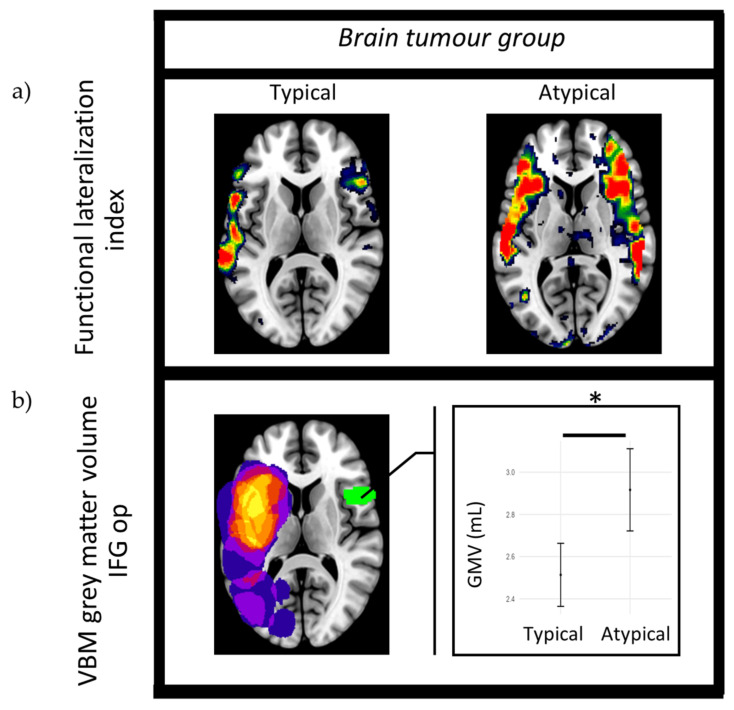
Brain tumor group: (**a**) functional language lateralization during the phonological association (PA) task for typical participant (subject 34) and atypical participant (subject 30). Higher activity (red) is observed in the left hemisphere of the typical participant, with high activity seen in both hemispheres in the atypical participant. (**b**) Differences in GMV between functionally typical and atypical participants during the PA task in the right opercular inferior frontal gyrus (IFG op). The lesion overlap map (purple/yellow) highlights the locations of participants’ tumors. * = *p* ≤ 0.05 after FDR correction.

During the SA and PA tasks, functional language lateralization was associated with GMV in seven ROIs following the FDR correction, as shown in Table 3. In these cases, the values were significantly higher in those with functional language lateralization as atypical (higher right hemisphere or bilateral fMRI activation) compared to typical participants. Those who were typical (higher left-hemisphere fMRI activation) displayed no larger GMV in comparison to the atypical group. The atypical participants displayed higher GMV values than the typical participants in the right MTG and SMG during the SA task, and the right IFG op (Figure 4a,b) IFG tri, STG, AG, and SMG during the PA task. No ROIs displayed larger GMV values in the functionally lateralized typical compared to atypical participants for any of the three tasks.

### 3.3. Logistic Regression

#### 3.3.1. Healthy Participants

**Figure 5:**
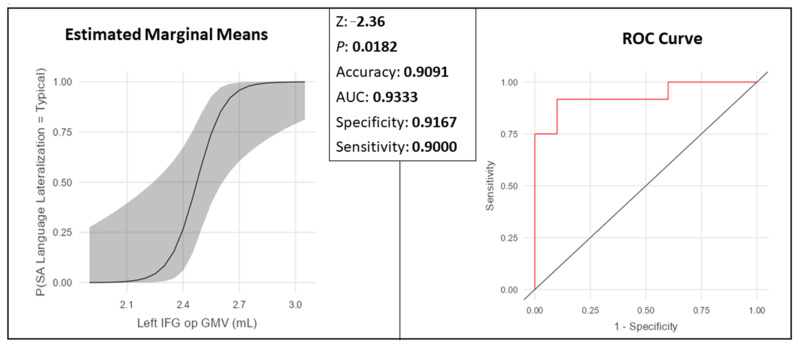
Left IFG op (Broca’s area). Logistic regression in healthy data group: as the GMV increases, the participant is more likely to be classified as functionally typical.

The left IFG op, which was significant in the linear regression following the FDR correction, remained significant in the binomial logistic regression, represented in Figure 5. Functional language lateralization was predicted as “typical” by a larger left IFG op GMV (Z = −2.36, *p* = 0.0182; accuracy, 90.91%, AUC = 0.9333 specificity = 91.67%, and sensitivity = 90.00%). 

#### 3.3.2. Left-Hemisphere Brain Tumor Participants

**Figure 6:**
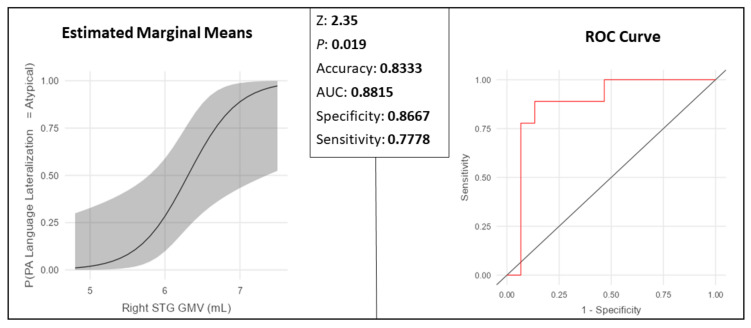
Right STG (Wernicke’s area). Logistic regression in the tumor patient group: as the GMV increases, the participant is more likely to be classified as functionally atypical.

All seven ROIs that appeared to be significant following the FDR correction in the linear regressions were also significant for the binomial logistic regressions, an example being the right STG as displayed in Figure 6. This region showed accuracy of 83.3%, AUC of 88.15%, sensitivity of 78.78%, and specificity of 86.67% for predicting the functional language lateralization of the participants.

## 4. Discussion

This study aimed to investigate the relationship between functional language lateralization and gray matter volume (GMV) in language-related regions of interest (ROIs), and to evaluate whether GMV can predict language lateralization as effectively as task-based fMRI. Our findings indicate that GMV can indeed serve as a supplementary tool for assessing language lateralization, with higher GMV values in specific ROIs correlating with typical and atypical language patterns.

Our results suggest that gray matter volume (GMV) in specific language regions can complement traditional fMRI for assessing language lateralization. In the healthy participants, typical left-hemisphere language dominance was predicted by higher GMV in the left inferior frontal gyrus (IFG-op). Conversely, in the brain tumor patients, atypical language lateralization was associated with increased GMV in the right-hemisphere regions. These results align with previous studies linking fMRI-derived language lateralization with GMV in the corresponding brain regions [20,30], and they are consistent with the well-documented phenomenon of neuroplasticity, where language functions often shift to the right hemisphere in response to damage or tumors in the left hemisphere [23,24,39]. Therefore, the increased GMV observed in the right hemisphere among those patients with atypical language lateralization supports the expected reorganization patterns and aligns with the existing research on brain plasticity [21]. However, the novelty of our study is that it displays GMV specifically in the language regions, of particular importance in presurgical tumor-removal planning. While our study replicates the findings of increased GMV in functionally dominant left hemispheres and contrasts with right dominance in right-dominant individuals, some regions like the supplementary motor area (SMA) and the IFG-orb showed no significant associations, possibly due to their broader functional roles and less direct involvement in language production [40,41,42].

Our results demonstrated variability in GMV associations with functional language lateralization across tasks, particularly in the tumor patients. The SA and PA tasks exhibited more GMV associations than the VG task. Specifically, the VG task did not produce any FDR-corrected GMV-fMRI associations, which was expected and is consistent with previous research highlighting the reliability of SA and PA tasks compared to VG tasks, particularly in patient groups with lesions [8,43]. Combining tasks can enhance sensitivity and accuracy in determining functional language lateralization [44,45,46]; however, in this study, fMRI activation’s link to GMV is superiorly displayed in the SA and PA tasks over the VG task rather than the tasks being assessed in a complementary manner. This potentially explains why the results between the functionally typical and atypical participants differed according to the task performed.

Analyzing the tumor participants posed challenges due to potential false negatives in fMRI signals near the brain tumor area [47], with GMV’s association with functional language lateralization paving the way for GMV as an alternative when fMRI is unreliable. Logistic regression was employed to address the need for classification algorithms in language research [21,48]. Future research may explore additional classification methods, such as support vector machines (SVMs), which have shown promise in other research areas [49,50].

A primary concern is the potential circularity in comparing GMV to fMRI. Since our method aims to be a complementary approach to fMRI, it is crucial to establish how it performs relative to fMRI in terms of accuracy and reliability [51]. Our results should be interpreted with the understanding that GMV cannot surpass fMRI’s performance in identifying language lateralization if it is dependent on the same underlying data despite it being able to provide underlying structural explanations of brain function [52]. To address this, we acknowledge that, while GMV offers a stable alternative, it cannot be considered superior to fMRI without direct comparisons using intra-operative cortical stimulation data or similar benchmarks. Future studies could benefit from incorporating such data to validate GMV’s effectiveness relative to fMRI.

This study has limitations. The ROI approach may miss potential effects in other regions, introducing user bias. A larger sample size across various tumor progression stages would provide further insights into patient care [44,45,46]. Furthermore, as a brain tumor can complicate the VBM calculation of tissue intensities during segmentation in the region surrounding the tumor, only right-hemisphere ROIs were included in this study in the brain tumor group [48]. It is important to acknowledge the small sample size of the current study due to the generally late diagnosis of brain tumors in the Chilean clinical context. Furthermore, while the rates of atypical language lateralization observed in the healthy participants may seem inconsistent with the established literature, which typically reports higher rates of left lateralization in right-handed individuals, we took several steps to ensure the robustness of our findings. Although we did not perform a formal cross-validation, we meticulously reviewed our data collection and analysis procedures to confirm their accuracy and consistency. To address the observed atypical lateralization, future research should investigate additional factors that may contribute to these findings and consider further validation methods to better understand the observed variability in language lateralization patterns.

## 5. Conclusions

Our results demonstrate that gray matter volume (GMV) in specific language regions can complement functional MRI (fMRI) for assessing language dominance, particularly when fMRI data are unavailable or unreliable. The study reveals that GMV measurements align with language lateralization patterns in both healthy participants and individuals with left-hemisphere tumors. While GMV shows potential as a supplementary tool in preoperative planning, its effectiveness as a standalone measure for language dominance remains to be validated against established benchmarks like intra-operative cortical stimulation. Future research should incorporate such benchmarks to confirm GMV’s clinical applicability and optimize its use in surgical decision-making. Although our study highlights GMV’s potential utility, further validation is necessary to fully establish its role in language lateralization assessment.

## Figures and Tables

**Table 1:** Information on participants. The healthy group and brain tumor group classified as typically or atypically lateralized based on the task being conducted. Age, TIV, and tumor volume are presented as mean values ± SD, with T and *p* values obtained from independent *t*-tests. Sex and handedness are presented as a male/female or left/right ratio, with *p* values obtained from independent tests of independence. * = significant *p* value < 0.05. *N* = number of participants in a particular group.

	Healthy Participant Group (*N* = 22)				Tumor Participant Group (*N* = 28)			
	PA task				PA task			
	Typical	Atypical	$T\;(x^{2}$)	*p*	Typical	Atypical	$T\;(x^{2}$)	*p*
N	11	11			15	9		
Age (years)	41.6 ± 14.7	41.1 ± 19.9	0.10	0.95	45 ± 14.6	42.1 ± 17.8	0.43	0.67
TIV (mL)	1498.0 ± 100.8	1386.9 ± 141.0	2.13	0.05 *	1403.8 ± 189.9	1374.3 ± 118.0	0.41	0.68
Tumor Volume (mL)	N/A	N/A	N/A	N/A	101.8 ± 166.7	48.1 ± 51.0	0.94	0.36
Sex (M/F)	6/4	4/7	0.73	0.39	4/11	5/4	2.00	0.16
Handedness (L/R)	4/7	2/9	0.92	0.34	0/15	1/8	1.74	0.19
	SA task				SA task			
	Typical	Atypical	$T\;(x^{2}$)	*p*	Typical	Atypical	$T\;(x^{2}$)	*p*
N	12	10			18	10		
Age (years)	42.8 ± 17.9	39.6 ± 16.8	0.42	0.68	44.0 ± 14.5	44.9 ± 18.0	−0.14	0.89
TIV (mL)	1481.3 ± 129.2	1395.9 ± 127.0	1.56	0.14	1387.5 ± 179.5	1417.3 ± 128.2	−0.46	0.65
Tumor Volume (mL)	N/A	N/A	N/A	N/A	95.5 ± 153.8	40.2 ± 46.3	1.1	0.28
Sex (M/F)	7/5	3/7	1.77	0.19	6/12	5/5	0.75	0.39
Handedness (L/R)	4/8	2/8	0.49	0.48	0/18	1/9	1.87	0.17
	VG task				VG task			
	Typical	Atypical	$T\;(x^{2}$)	*p*	Typical	Atypical	$T\;(x^{2}$)	*p*
N	14	8			17	7		
Age (years)	43.5 ± 16.2	37.5 ± 18.9	0.79	0.44	44.4 ± 16.4	42.9 ± 14.4	0.21	0.84
TIV (mL)	1432.7 ± 119.4	1459.5 ± 160.0	−0.45	0.66	1384.7 ± 183.4	1412.4 ± 115.0	−0.37	0.72
Tumor Volume (mL)	N/A	N/A	N/A	N/A	90.3 ± 156.7	60.8 ± 69.3	0.47	0.64
Sex (M/F)	5/9	5/3	1.47	0.22	7/10	2/5	0.34	0.56
Handedness (L/R)	3/11	3/5	0.66	0.42	0/17	1/6	2.53	0.11

**Table 2:** Linear regression results between LI and GMV in healthy participants (controlled for age and TIV). Each line represents an ROI where the GMV was significantly predicted by LI. * = significant after false discovery rate (FDR) correction (FDR of 0.05).

Healthy Participant GMV-fMRI Associations—Typical > Atypical				
ROI	Hemisphere	Task	t	*p*
Opercular inferior frontal gyrus	L	SA	−4.55	0.00002 *
Supplementary motor area	R	SA	−2.22	0.04
Orbital inferior frontal gyrus	R	PA	−2.16	0.045
Supramarginal gyrus	R	VG	−2.96	0.0082
Supramarginal gyrus	L	VG	−2.47	0.024
Superior temporal gyrus	R	VG	−2.50	0.022
Superior temporal gyrus	L	VG	−2.25	0.038
Angular gyrus	L	VG	−2.18	0.043

**Table 3:** Linear regression results between LI and GMV in participants with a left-hemisphere brain tumor (controlled for age, TIV, and tumor volume). Each line represents an ROI where the GMV was significantly predicted by LI. * = significant after false discovery rate (FDR) correction (FDR of 0.05).

Tumor Participant GMV-fMRI Associations—Atypical > Typical				
ROI	Hemisphere	Task	t	*p*
Medial temporal gyrus	R	SA	2.62	0.015 *
Supramarginal gyrus	R	SA	2.90	0.008 *
Opercular inferior frontal gyrus	R	SA	2.09	0.048
Superior temporal gyrus	R	SA	2.48	0.021
Angular gyrus	R	SA	2.42	0.024
Opercular inferior frontal gyrus	R	PA	3.38	0.003 *
Triangular inferior frontal gyrus	R	PA	2.72	0.014 *
Superior temporal gyrus	R	PA	2.96	0.008 *
Angular gyrus	R	PA	3.01	0.007 *
Supramarginal gyrus	R	PA	2.80	0.011 *

## Data Availability

The datasets containing sensitive patient clinical data, as presented in this article, are not currently accessible. For inquiries, including requests for datasets and raw data, please contact the corresponding author directly. Requests to access the datasets can be directed to Carolina Mendez at carolinamendez@me.com.

## Supplementary Materials

The following supporting information can be downloaded at https://www.mdpi.com/article/10.3390/brainsci14100954/s1, Figure S1: fMRI tasks. Figure S2: Language regions of interest. Table S1: Tumor grade, location, and volume, as well as further demographic data of the brain tumor group.
