# Supplementary material for: Assessing Language Lateralization through Gray Matter Volume: Implications for Preoperative Planning in Brain Tumor Surgery
Source: Brain Sci. 2024 Sep 24;14(10):954. doi: 10.3390/brainsci14100954 (PMC11506207; doi:10.3390/brainsci14100954)
Supplement: Supplementary file 1 [file brainsci-14-00954-s001.zip › Supplementary Figures.pdf]

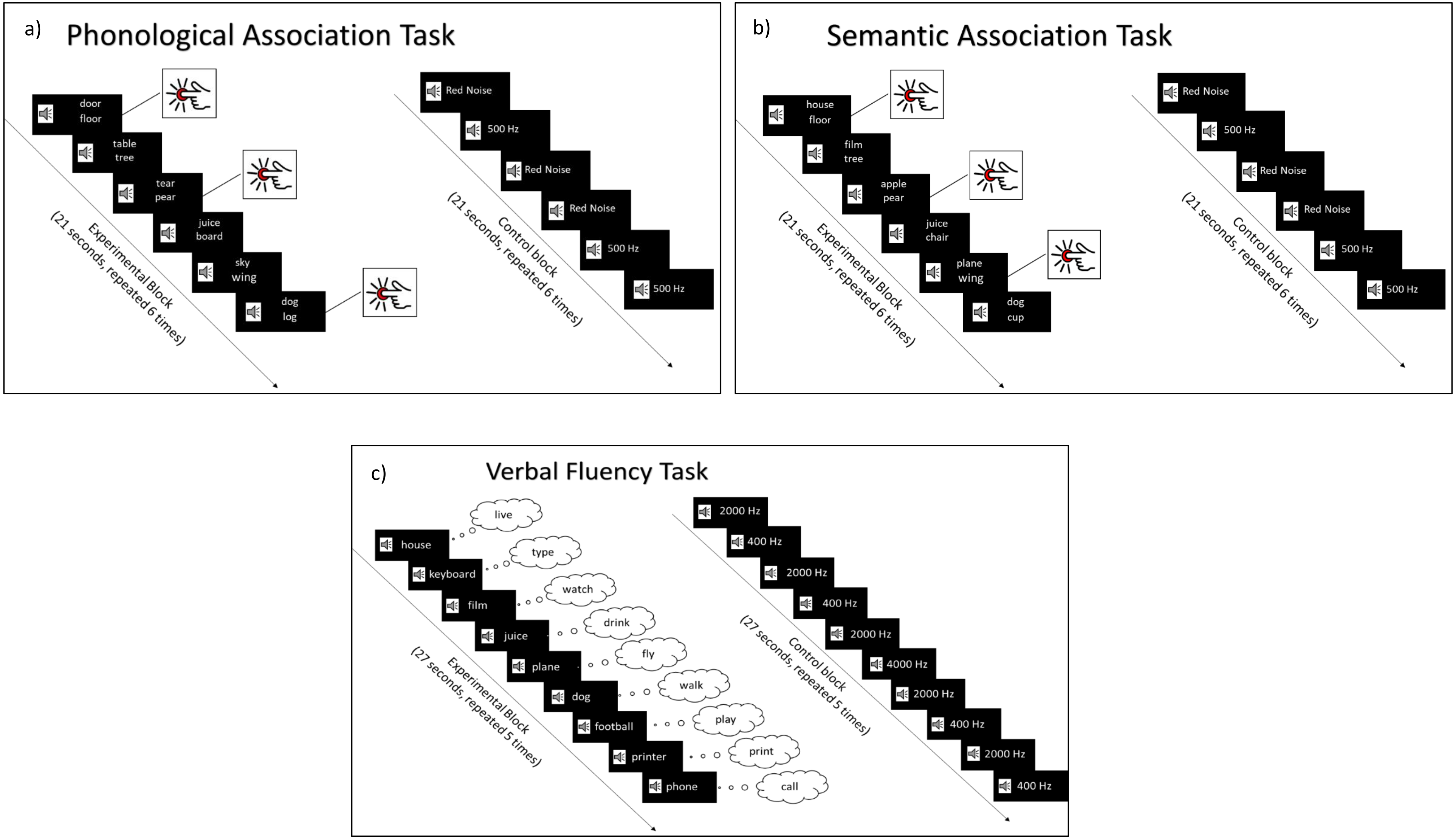


**Supplementary Figure S1.**

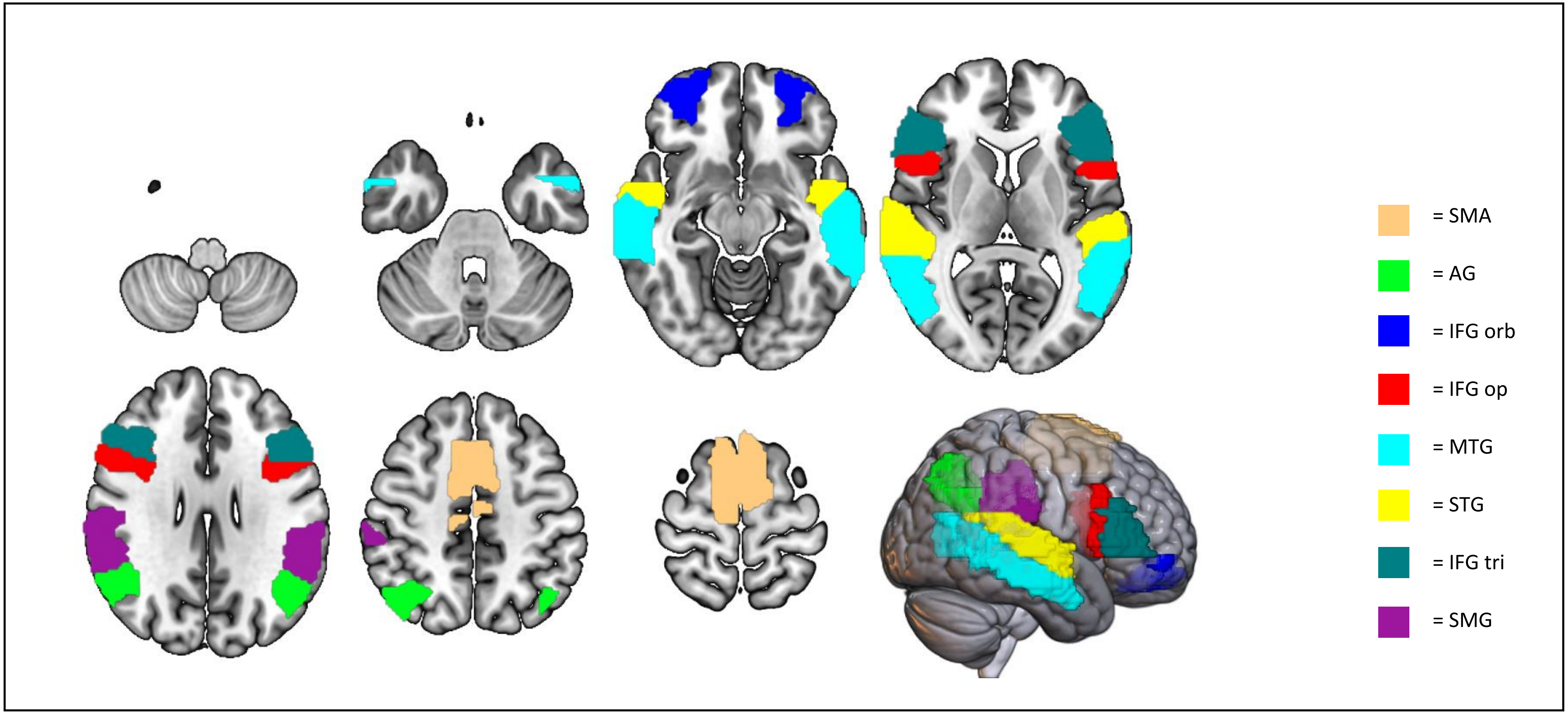


**Supplementary Figure S2.**

| Brain tumour group | | | | | | |
|---|---|---|---|---|---|---|
| No. | Sex | Age | Handedness | Tumour Volume | WHO classification | Tumour location |
| **1** | F | 61 | R | 186.41 | IV | OCCIPITAL |
| **2** | M | 52 | R | 29.25 | IV | TEMPORAL |
| **3** | M | 58 | R | 3.92 | N/A | PARIETAL-OCCIPITAL |
| **4** | M | 65 | R | 38.53 | IV | TEMPORAL |
| **5** | M | 57 | R | 7.26 | N/A | TEMPORAL-MEDIAL |
| **6** | F | 31 | R | 118.64 | N/A | FRONTAL-TEMPORAL |
| **7** | F | 55 | R | 4.8 | N/A | FRONTAL-PARIETAL |
| **8** | M | 78 | R | 4.91 | N/A | PARIETAL |
| **9** | F | 43 | R | 68.24 | III | FRONTAL-TEMPORAL |
| **10** | F | 38 | R | 21 | II | PARIETAL-OCCIPITAL |
| **11** | F | 38 | L | 1.04 | N/A | FRONTAL-INFERIOR |
| **12** | F | 34 | R | 1.32 | N/A | FRONTAL-SUPERIOR |
| **13** | F | 39 | R | 475.77 | N/A | TEMPORAL-INSULAR |
| **14** | F | 31 | R | 47.08 | II | PARIETAL |
| **15** | M | 48 | R | 24.94 | II | FRONTAL-MEDIIAL |
| **16** | M | 29 | R | 139.43 | N/A | TEMPORAL-MEDIAL |
| **17** | F | 37 | R | 221.18 | N/A | FRONTAL-TEMPORAL |
| **18** | F | 55 | R | 2.35 | N/A | PARIETAL |
| **19** | F | 40 | R | 17.23 | N/A | FRONTAL-SUPERIOR |
| **20** | F | 42 | R | 1.75 | II | FRONTAL-SUPERIOR |
| **21** | F | 33 | R | 44.48 | II | FRONTAL-INFERIOR |
| **22** | F | 31 | R | 1.18 | N/A | TEMPORAL-INFERIOR |
| **23** | F | 30 | R | 3.02 | N/A | FRONTAL-SUPERIOR |
| **24** | M | 20 | R | 16.91 | N/A | FRONTAL-PARIETAL |
| **25** | M | 25 | R | 38.03 | IV | FRONTAL-INFERIOR |
| **26** | M | 76 | R | 4.15 | N/A | TEMPORAL-MEDIAL |
| **27** | F | 30 | R | 479.22 | N/A | TEMPORAL |
| **28** | M | 65 | R | 118.53 | IV | FRONTAL-INFERIOR |

**Supplementary Table S1.**
